# Autophagy favors survival of corpora lutea during the long-lasting pregnancy of the South American plains vizcacha, *Lagostomus maximus* (Rodentia, Caviomorpha)

**DOI:** 10.1038/s41598-024-61478-5

**Published:** 2024-05-16

**Authors:** Daira A. Caram, Pablo I. F. Inserra, Alfredo D. Vitullo, Noelia P. Leopardo

**Affiliations:** 1 Centro de Estudios Biomédicos, Básicos, Aplicados y Desarrollo (CEBBAD), Universidad Maimónides, Ciudad Autónoma de Buenos Aires, Argentina; 2 Consejo Nacional de Investigaciones Científicas y Técnicas (CONICET), Buenos Aires, Argentina

**Keywords:** Macroautophagy, Animal physiology

## Abstract

The corpus luteum (CL) is a transient endocrine gland that plays a crucial role in establishing and maintaining pregnancy. Although autophagy and apoptosis have been suggested as cooperative mechanisms, their interaction within the CL of pregnant mammals has not been thoroughly investigated. To understand the collaborative function of autophagy and apoptosis in the CL, we analyzed both mechanisms during pregnancy in the South American plains vizcacha, *Lagostomus maximus*. This rodent undergoes a decline in progesterone levels during mid-gestation, a reactivation of the hypothalamus-hypophysis-gonadal axis, and the incorporation of new functional secondary CL. Our analysis of autophagy markers BECLIN 1 (BECN1), SEQUESTOSOME1 (SQSTM1), Microtubule-associated protein light chain 3 (LC3B), and lysosomal-associated membrane protein 1 (LAMP1) and anti- and pro-apoptotic markers BCL2 and ACTIVE CASPASE 3 (A-C3) revealed interactive behaviors between both processes. Healthy primary and secondary CL exhibited positive expression of BECN1, SQSTM1, LC3B, and LAMP1, while regressed CL displayed enhanced expression of these autophagy markers along with nuclear A-C3. Transmission electron microscopy revealed a significant formation of autophagic vesicles in regressed CL during full-term pregnancy, whereas healthy CL exhibited a low number of autophagy vesicles. The co-localization between LC3B and SQSTM1 and LC3B with LAMP1 was observed in both healthy and regressed CL during pregnancy, while co-localization of BECN1 and BCL2 was only detected in healthy CL. LC3B and ACTIVE CASPASE 3 co-localization were detected in a subset of luteal cells within the regressing CL. We propose that autophagy could act as a survival mechanism in the CL, allowing the pregnancy to progress until full-term, while also serving as a mechanism to eliminate remnants of regressed CL, thereby providing the necessary space for subsequent follicular maturation.

## Introduction

The corpus luteum (CL) is a transient endocrine gland that synthesizes both peptide and steroid hormones, serving as the primary source of progesterone (P4) during estrous/menstrual cycles. It is crucial for the establishment and maintenance of pregnancy^1^. The CL consists of distinct cell types, primarily steroidogenic large and small luteal cells, which originate from the wall of the postovulatory follicle^2^. The lifespan of the CL is determined by a balance between the presence and action of luteotropic and luteolytic factors^3^. At a certain period of the estrous cycle and pregnancy, the CL undergoes regression characterized by disruption of P4 synthesis (functional luteolysis) followed by morphological degeneration (structural luteolysis)^4^. The process is characterized by cytoplasmic vacuolization, lysosomes, accumulation of autophagosomes, and steroidogenic luteal cell apoptosis induced by autophagy^5^. Despite recent progress within the past few years, the intracellular mechanisms controlling luteal function and regression remain largely unknown^1^. It is commonly accepted that apoptosis underlies the process of structural regression, which ultimately leads to luteal cell death^6–8^. Research in rodents^8,9^, cows^10,11^, and pigs^12^ suggest that autophagy participates in CL regression. However, the full contribution of autophagy to the physiological function of the CL requires further elucidation. The CL serves as an excellent paradigm for examining the mechanisms that regulate cell survival and programmed cell death under physiological conditions^13–15^, as it is a structure that is generated and degenerated in a short period.

Autophagy is a cellular process that has been conserved throughout evolution and involves the degradation of abnormal proteins, organelles, and aggregates via autophagosomes^16–19^. This process is crucial for normal development, tissue and organ remodeling, as well as cell survival and death^20–22^. Some studies have provided evidence that autophagy is a pathway of lysosome-mediated degradation that involves the fusion of double-membrane autophagosomes with lysosomes, known as macroautophagy^16^. This entails the sequestration and transportation of cytoplasmic material to the lysosome for degradation and recycling^21^. It is worth noting that basal levels of autophagy are fundamental in maintaining cellular homeostasis under normal conditions^22–24^. Autophagy can be induced by various environmental stressors such as nutrient deficiency^25^, hypoxia^26^, hormonal alterations^27,28^, DNA or organelle damage^29,30^, amino acid deprivation^31,32^, exposure to high temperatures^33^, or even reactive oxygen species^34^. When the stimulus is heightened or if the damage remains unresolved, autophagy can lead to cell death or activate apoptosis^35,36^. Thus, depending on cellular and environmental conditions, autophagy can either promote cell death or protect cells from various types of injuries^37^. In light of this, apoptosis and autophagy have been proposed as cooperative mechanisms. However, their interaction within the CL of pregnant mammals has not been thoroughly investigated^38^. To enhance our understanding of the potential cooperative role of autophagy and apoptosis in CL regression and/or survival, we analyzed both mechanisms using the emerging seasonal breeding rodent model, *Lagostomus maximus*, commonly known as the South American plains vizcacha. The vizcacha is a fossorial hystricognath rodent primarily distributed in the Pampa plains of Argentina^39^. This species displays an exceptionally prolonged gestation period of five months^40,41^, in contrast to the very short gestation period of 20 days observed in their distant murine counterparts. Furthermore, pregnant vizcachas exhibit a biphasic P4 profile^40,42^ instead of the typical steady-increase P4 curve observed in most pregnant mammals^43^. Additionally, during early pregnancy, placental calcification and the thinning of uterine segmental arteries, as they move away from the cervix towards the ovary, strongly suggest that the vizcacha placenta is likely deficient in steroidogenesis^42–45^. As a result of the decrease in circulating P4 levels around day 90 of gestation, and the subsequent removal of the negative feedback on gonadotropin release, a new set of secondary CL emerges in mid-gestation^40,46–48^. These additional CLs have been precisely attributed to the recovery of P4 levels necessary for successful embryonic development and the completion of pregnancy^40^. However, there have been limited instances of luteal regression reported during the gestational period of the vizcacha. This suggests that the primary CL persists even though a new set of secondary CL has emerged^49,50^.

In this report, we present our analysis of the main markers of autophagy and apoptosis in the ovary of pregnant vizcachas. Our findings indicate limited interactivity between these processes, whereby autophagy promotes cell survival or cell death, contingent upon the analyzed structure. Our observations suggest that autophagy may serve as a mechanism for promoting cell survival within healthy CL during pregnancy. Conversely, autophagy appears to mediate the elimination of a small subset of regressed CL, with minimal involvement of apoptosis, during term pregnancy.

## Results

A significant number of primary CL was detected at early-pregnancy across the ovarian tissue. At this gestational stage, neither secondary CL (i.e., CL showing a retained oocyte) nor CL with signs of regression were detected (Table 1). At mid-gestation, the number of primary CL remained almost unchanged, secondary CL were first detected alongside a few CL with signs of regression (Table 1). At term-gestation, there was no significant variation in the number and size of secondary corpus luteum. However, it was observed that the number of primary corpus luteum was significantly lower with a smaller size, and a notable increase in the quantity of regressed corpus luteum was detected (Table 1).

**Table 1:** Semi-quantification of the number and major axis length of primary, secondary, and regressed corpora lutea (CL) of the vizcacha throughout pregnancy.

Stage of pregnancy	Primary CL		Secondary CL		Regressed CL	
	Mean number of CL (per mm^2^)	Mean CL mayor axis (µm)	Mean number of CL (per mm^2^)	Mean CL mayor axis (µm)	Mean number of CL (per mm^2^)	Mean CL mayor axis (µm)
Early	2.14 ± 0.57^ab^	280.03 ± 15.89^a^	ND	ND	ND	ND
Mid	2.49 ± 0.43^a^	317.61 ± 22.50^a^	0.78 ± 0.21^a^	222.05 ± 43.60^a^	0.21 ± 0.17^a^	165.21 ± 7.39^a^
Term	1.46 ± 0.36^b^	184.41 ± 32.18^b^	0.72 ± 0.27^a^	184.8 ± 35.81^a^	0.63 ± 0.19^b^	105.15 ± 38.46^b^

Data are expressed as mean ± standard deviation. Superscript letters in each column indicate significant differences (*p* < 0.05) throughout pregnancy. One-way ANOVA with the Bonferroni post hoc test was used for multiple comparisons. ND: not detected.

### Autophagy-related proteins in the corpora lutea of pregnant vizcacha

Autophagy-associated proteins BECN1, LC3B, and SQSTM1 were assessed in corpora lutea by means of cellular immunolocalization, protein molecular detection, and electron microscopy. BECN1 plays a crucial role in the formation of the pre-autophagosome structure. During autophagy induction, LC3B is cleaved or converted from LC3B-I to LC3B-II. LC3B-II then localizes to isolated membranes and autophagosome membranes, indicating the occurrence of autophagy. Furthermore, the amount of LC3B-II is positively correlated with the number of autophagosomes^23^. SQSTM1, on the other hand, functions as a selective autophagy receptor while also performing various functions in the ubiquitin–proteasome system, cellular metabolism, signaling, and apoptosis. This multifunctional, multi-domain adaptor protein is present in the autophagosome membranes^51^.

**Figure 1:**
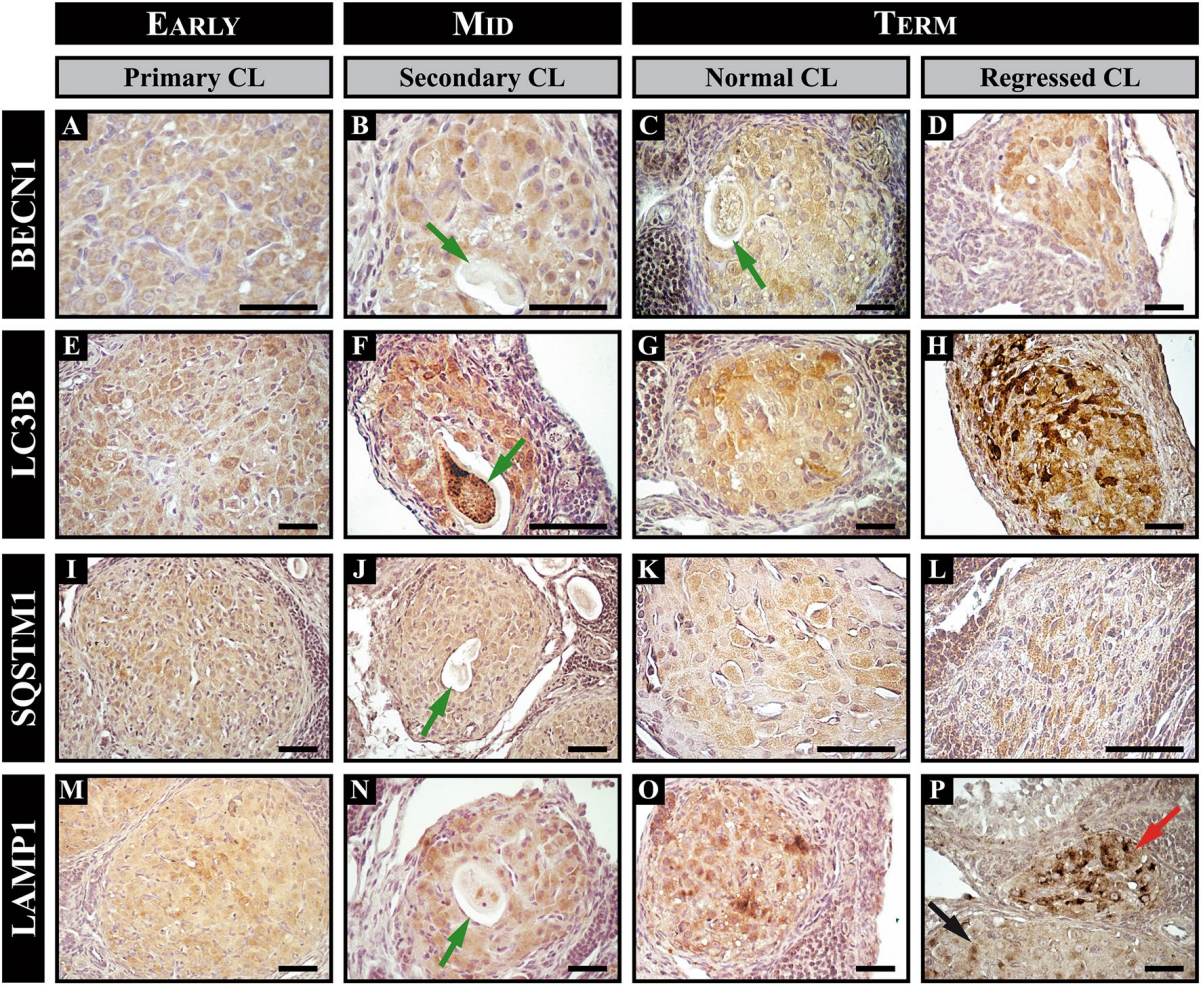
Immunolocalization of autophagy-related proteins in corpora lutea (CL) of the vizcacha throughout pregnancy. Immunostaining of BECN1 (**A**–**D**), LC3B (**E**–**H**), SQSTM1 (**I**–**L**), and LAMP1 (**M**–**P**) in primary (black arrow), secondary [note the retained oocyte (green arrows)], and regressed CL (red arrow) in early-, mid-, and term-pregnancy. Normal CL refers to healthy primary or secondary CL with no morphological evidence of regression. Low magnification images illustrating the expression of each autophagic protein throughout gestation are displayed in the supplementary material Fig. S1. Scale bars: 50 μm.

**Figure 2:**
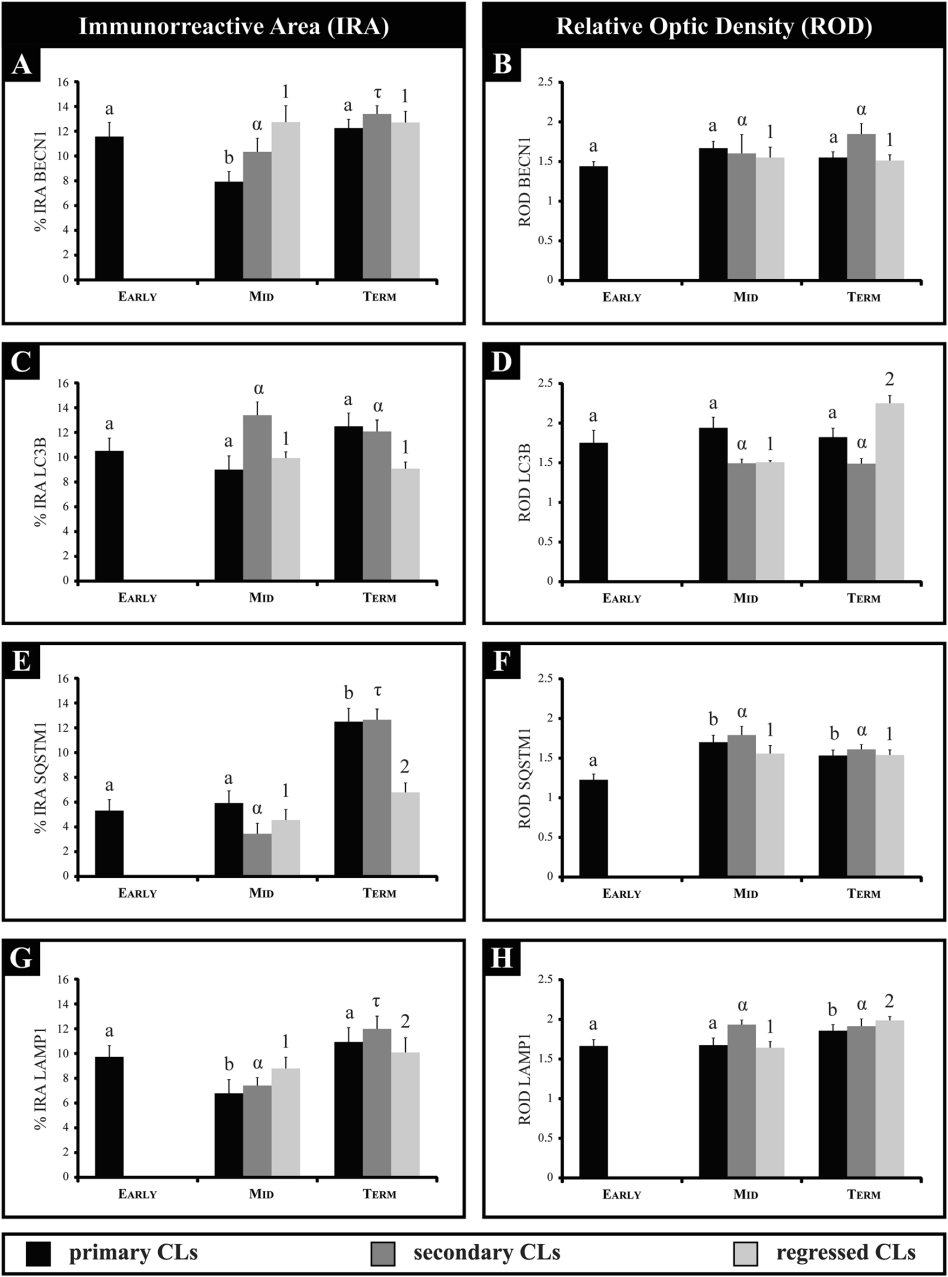
Quantification of relative optical density (ROD) and immunoreactive area (IRA) of autophagy-related proteins in primary, secondary, and regressed corpora lutea (CL) of the vizcacha throughout pregnancy. IRA and ROD of BECN1 (**A**–**B**), LC3B (**C**–**D)**, SQSTM1 (**E**–**F**), and LAMP1 (**G**–**H**). Values are expressed as mean ± standard deviation. Different Roman letters indicate significant IRA & ROD differences of primary CLs among groups; different Greek letters indicate significant IRA & ROD differences of secondary CLs among groups; and different numbers indicate significant IRA & ROD differences of regressed CLs among groups (*p* < 0.05).One-way ANOVA with the Bonferroni post hoc test was used for multiple comparisons.

**Figure 3:**
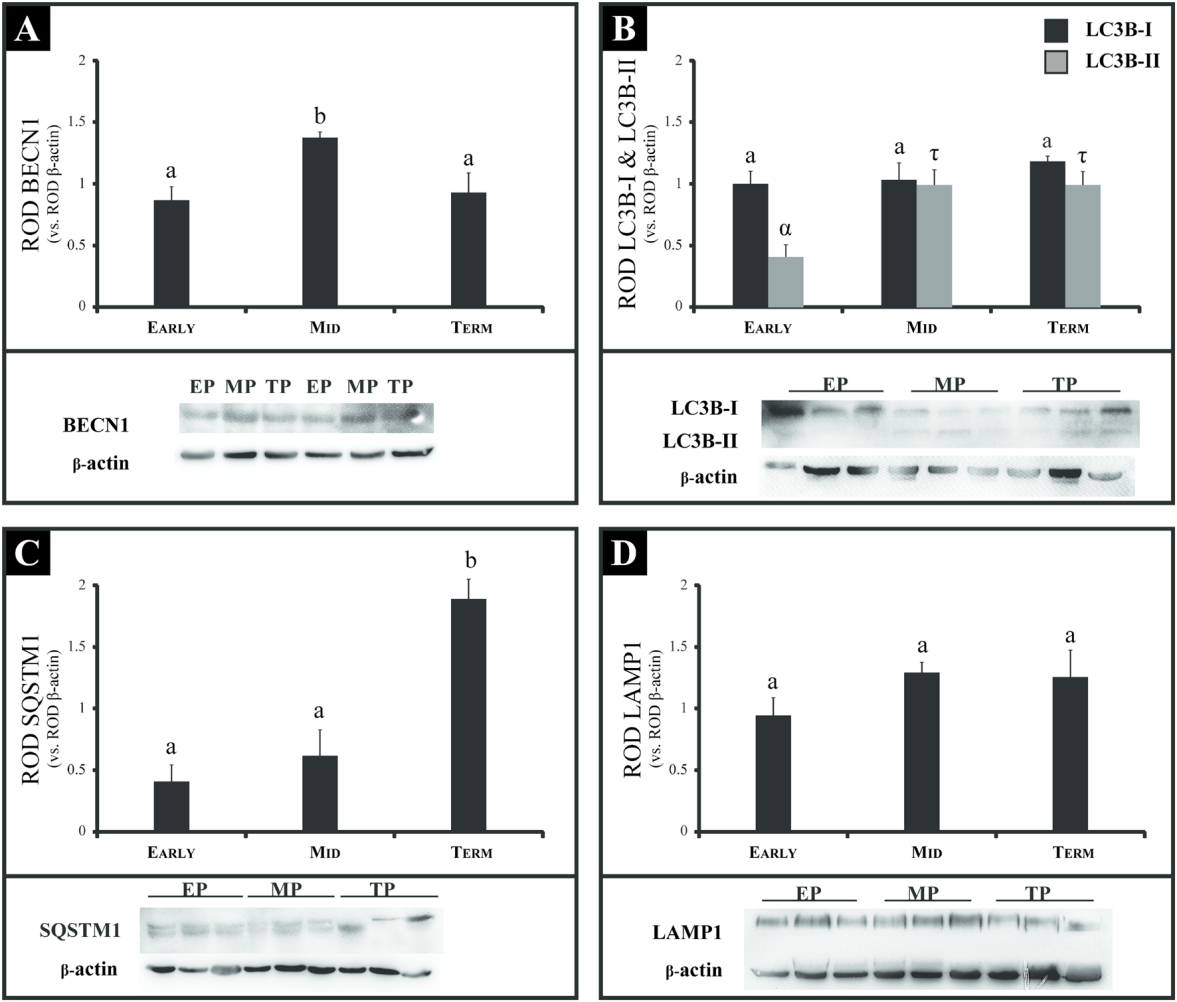
Quantification of autophagy-related protein expression in the ovary of the vizcacha throughout pregnancy. Relative optical density (ROD) quantification of BECN1 (**A**), LC3B-I & LC3B-II (**B**), SQSTM1 (**C**), and LAMP1 (**D**). β-actin ROD expression was used as a normalizer protein. Representative images of immunoblots for BECN1, LC3B-I, LC3B-II, SQSTM1, LAMP1, and β-actin are shown. Values are expressed as mean ± standard deviation. Different Roman letters indicate significant ROD differences among groups. In the case of LC3B, different Roman letters indicate significant ROD differences of LC3B-I among groups, and different Greek letters indicate significant ROD differences of LC3B-II among groups (*p* < 0.05). One-way ANOVA with the Bonferroni post hoc test was used for multiple comparisons. The blots were cut before hybridization with indicated primary antibodies to prevent band overlap. ACTIN was revealed onto a distinct membrane owing to its closely associated molecular weights with BECLIN1 and SQSTM1. In some cases, membrane edges are unclear due to the high signal-to-noise ratio of luminescence intensity. Original blots are presented in Supplementary Fig. S3.

During early and mid-pregnancy, BECN1 was localized within the cytoplasm of luteal cells exhibiting weak immunostaining (Fig. 1, Fig. S1). However, at term-pregnancy, strong BECN1 immunostaining was detected in both healthy and regressed CL (Fig. 1, Fig. S1). The examination of BECN1-positive CL indicates that more than 72% exhibit positive staining for this protein, and the number of CL expressing BECN1 rises as gestation progresses (Table 2). The IRA for BECN1 exhibited variations during different stages of gestation. It is noteworthy that in the primary CL, the IRA for BECN1 significantly decreased at mid-pregnancy, while the ROD remained constant throughout gestation (Fig. 2A, B). In the secondary CL, there was a significant increase in the IRA for BECN1 from mid- to term-pregnancy; however, the ROD showed no significant differences throughout gestation (Fig. 2A, B). In the regressed CL, there were no statistically significant differences in IRA and ROD values throughout gestation (Fig. 2A, B). Moreover, Western blot analysis evinced a significant rise in BECN1 levels during mid-pregnancy when compared to the other groups (Fig. 3A).

**Table 2:** Autophagy-related proteins and ACTIVE CASPASE 3 immunodetection in corpora lutea (CL) of pregnant vizcacha.

	Mean percentage of positive CL for BECN1		
Stage of pregnancy	Primary CL	Secondary CL	Regressed CL
Early	76.30 ± 12.41^a^	ND	ND
Mid	81.82 ± 7.91^a^	83.92 ± 10.03^a^	72.26 ± 6.17^a^
Term	86.63 ± 2.69^a^	87.16 ± 7.50^a^	87.41 ± 6.18^b^

	Mean percentage of positive CL for LC3B		
Stage of pregnancy	Primary CL	Secondary CL	Regressed CL
Early	72.71 ± 4.13^a^	ND	ND
Mid	87.16 ± 6.23^b^	86.83 ± 3.34^a^	84.11 ± 7.97^a^
Term	84.35 ± 5.17^b^	84.18 ± 8.12^a^	90.46 ± 3.19^a^

	Mean percentage of positive CL for SQSTM1		
Stage of pregnancy	Primary CL	Secondary CL	Regressed CL
Early	8.26 ± 1.09^a^	ND	ND
Mid	69.51 ± 13.47^b^	69.29 ± 6.21^a^	51.35 ± 11.03^a^
Term	65.14 ± 3.87^b^	68.89 ± 2.77^a^	68.16 ± 9.22^a^

	Mean percentage of positive CL for LAMP1		
Stage of pregnancy	Primary CL	Secondary CL	Regressed CL
Early	77.90 ± 9.11^a^	ND	ND
Mid	67.73 ± 9.92^a^	73.32 ± 7.41^a^	76.18 ± 4.36^a^
Term	84.45 ± 3.74^a^	85.04 ± 10.30^a^	87.61 ± 5.95^b^

	Mean percentage of positive CL for A-C3		
Stage of pregnancy	Primary CL	Secondary CL	Regressed CL
Early	18.52 ± 8.06^a^	ND	ND
Mid	24.32 ± 3.19^a^	76.38 ± 6.19^a^	14.94 ± 3.05^a^
Term	7.94 ± 1.89^b^	91.30 ± 10.01^b^	22.73 ± 4.81^b^

Values are expressed as mean percentage ± standard deviation. Different letters in each column indicate significant differences (*p* < 0.05) for each protein throughout pregnancy. One-way ANOVA with the Bonferroni post hoc test was used for multiple comparisons. A-C3: ACTIVE CASPASE 3; BECN1: BECLIN 1; SQSTM1: SEQUESTOSOME 1; ND: not detected.

LC3B immuno-localized in the cytoplasm of the luteal cells from both primary and secondary CL. A majority of CL, greater than 73%, exhibited positive staining for LC3B. Furthermore, it was observed that the proportion of CL expressing LC3B tended to increase as gestation progressed (Table 2). Primary and secondary Clawer positive during pregnancy (Fig. 1E–G, Fig. S1). There were no significant differences observed in the IRA and ROD of LC3B throughout gestation for primary and secondary CL (Fig. 2C, D). In regressed CL, the IRA did not show any significant differences, but ROD did exhibit a significant rise toward the end of pregnancy (Fig. 2C, D).

Microscopic examination revealed that there were two distinct modes of LC3B expression. LC3B-I exhibited a dispersed distribution across the cytoplasm, while LC3B-II was associated with punctuate structures within the cytoplasm (Fig. 1E–H, Fig. S1). Both patterns were discerned in luteal cells; however, the punctuate structures in the oocytes of secondary CL and regressed CL were more prominent (Fig. 1F, Fig. S1). Significantly elevated levels of LC3B-I and LC3B-II were detected during term pregnancy via protein expression analysis of LC3B (Fig. 3B).

SQSTM1 localized in the cytoplasm of luteal cells. Quantitative analysis revealed that only a small percentage (8%) of primary CL exhibited positive staining for SQSTM1 at early-pregnancy, but the majority of primary CL (> 51%) were positive for this protein at mid- and term-pregnancy (Table 2). SQSTM1 was immunolocalized in primary CL during pregnancy (Fig. 1I, K, Fig. S1) and significant differences in the IRA were observed at term-pregnancy (Fig. 2E). On the other hand, the ROD of SQSTM1 for primary CL showed a significant increase from mid- to term-pregnancy (Fig. 2F). SQSTM1 was also positive in secondary CL during pregnancy (Fig. 1J, Fig. S1). More than 68% of the secondary CLs were positive for this protein from mid-gestation. Significant differences in the IRA were observed at term-pregnancy, while no significant differences were observed in the ROD (Fig. 2E, F). In regressed CL, protein expression of SQSTM1 was found to be heterogeneous within the structure (Fig. 1L, Fig. S1). More than 58% of the regressed CLs were positive for the protein from mid-gestation. No significant differences were observed in the ROD, but the IRA increased significantly at the end of pregnancy (Fig. 2E, F). Western blot analysis showed two isoforms of SQSTM1, SQSTM1-H1 (full-length isoform) and SQSTM1-H2 (partly devoid of the PB1 domain). SQSTM1-H1 showed a significant increase at the end of pregnancy compared to the other groups (Fig. 3C).

Finally, we evaluated the expression of LAMP1, the membrane constituent of functional lysosomes. LAMP1 immunolocalized in the cytoplasm of luteal cells (Fig. 1, Fig. S1); the majority of CLs exhibited positive staining for this protein, exceeding 68%, and the number of LAMP1-expressing CL tended to increase as gestation progressed (Table 2). The expression of LAMP1 was observed in primary, secondary, and regressed CL throughout pregnancy (Fig. 1M–P, Fig. S1). Significant differences were observed in the IRA and ROD of LAMP1 throughout gestation for primary and regressed CL (Fig. 2G, H). In secondary CL, no significant differences were observed in the ROD, but the IRA increased significantly towards the end of pregnancy (Figs. 1N and 2G, H; Fig. S1). Strong immunostaining of LAMP1 was observed in regressed CL compared to healthy ones (Fig. 1P, Fig. S1). Western blot analysis showed a significant increase in LAMP1 expression from mid- to term-pregnancy (Fig. 3D).

### Autophagic flux analysis in the ovary of pregnant vizcacha

**Figure 4:**
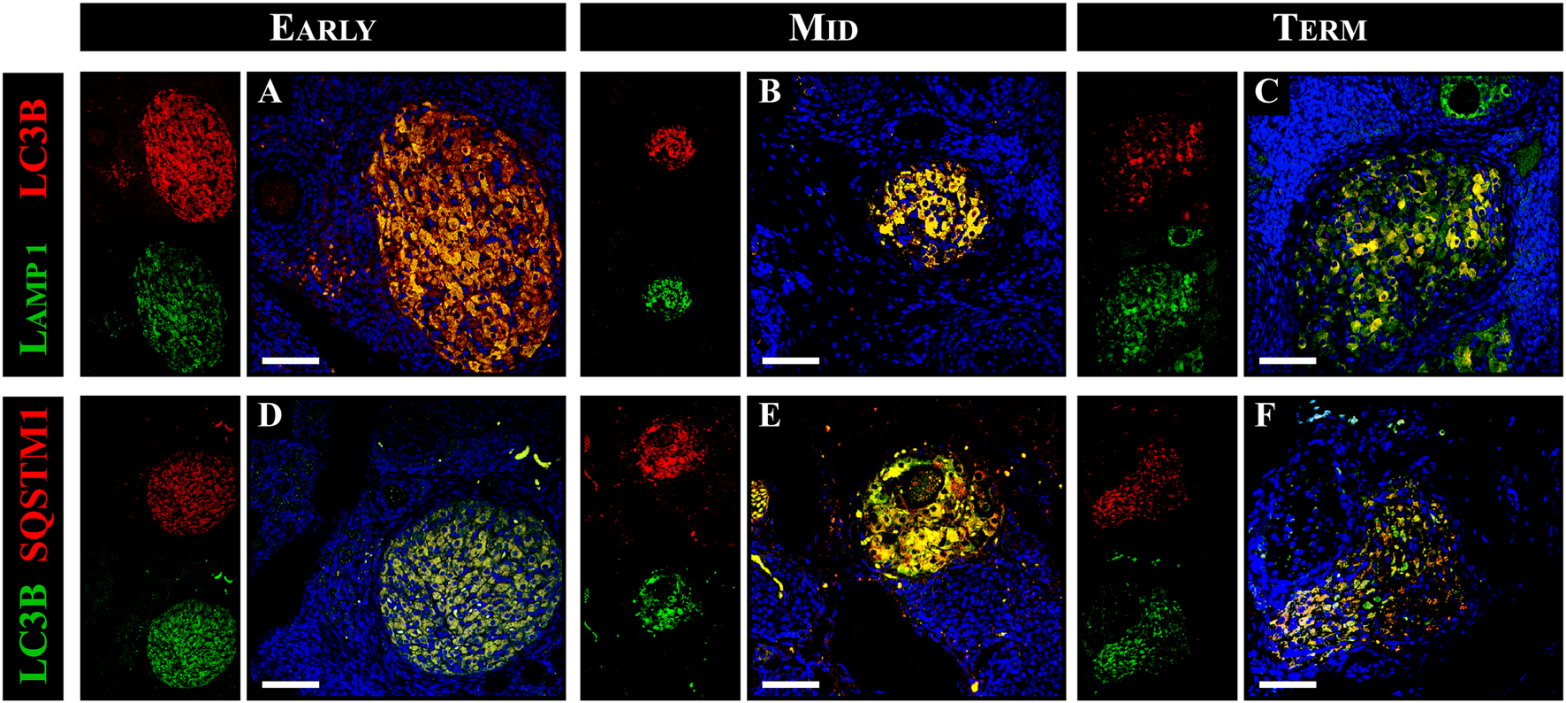
Colocalization of LC3B with LAMP1 and SQSTM1 in healthy and regressed corpora lutea (CL) of the vizcacha throughout pregnancy. Merged images of LC3B (cytoplasmic red staining) and LAMP1 (cytoplasmic green staining) in primary CL in early-pregnancy (**A**), secondary CL in mid-pregnancy (**B**), and regressed CL in term-pregnancy **(C)**. Merged images of LC3B (cytoplasmic red staining) and SQSTM1 (cytoplasmic green staining) in primary CL in early-pregnancy (**D**), secondary CL in mid-pregnancy **(E),** and regressed CL in term-pregnancy (**F**). Yellow cytoplasmic staining indicates colocalization of LC3B with LAMP1 or with SQSTM1 in luteal cells of primary (**A** and **D**), secondary (**B** and **E**), and regressed CL (**C** and **F**). All images show DAPI nuclear staining (blue). Scale bar: 50 μm.

Since autophagic flux is characterized by the dynamic process of autophagosome generation, their fusion with lysosomes, and the subsequent degradation of autophagic substrates in autolysosomes^17^, we analyzed the colocalization of the autophagic substrates LC3B/SQSTM1 (autophagosome formation) and LC3B/LAMP1 (fusion with lysosomes) to confirm that these proteins were within the same cell as an indicator of the autophagic flux. We observed that LC3B colocalized with SQSTM1 in the majority of luteal cells of primary, secondary, and regressed CL throughout pregnancy (Fig. 4A–C). On the other hand, LC3B colocalized with LAMP1 in primary CL and secondary CL luteal cells, while in regressed CL, LAMP1 expression was observed in most luteal cells with a concomitant decrease in LC3B expression (Fig. 4D–F).

### Ultrastructural features of autophagy in the corpora lutea throughout vizcacha pregnancy

**Figure 5:**
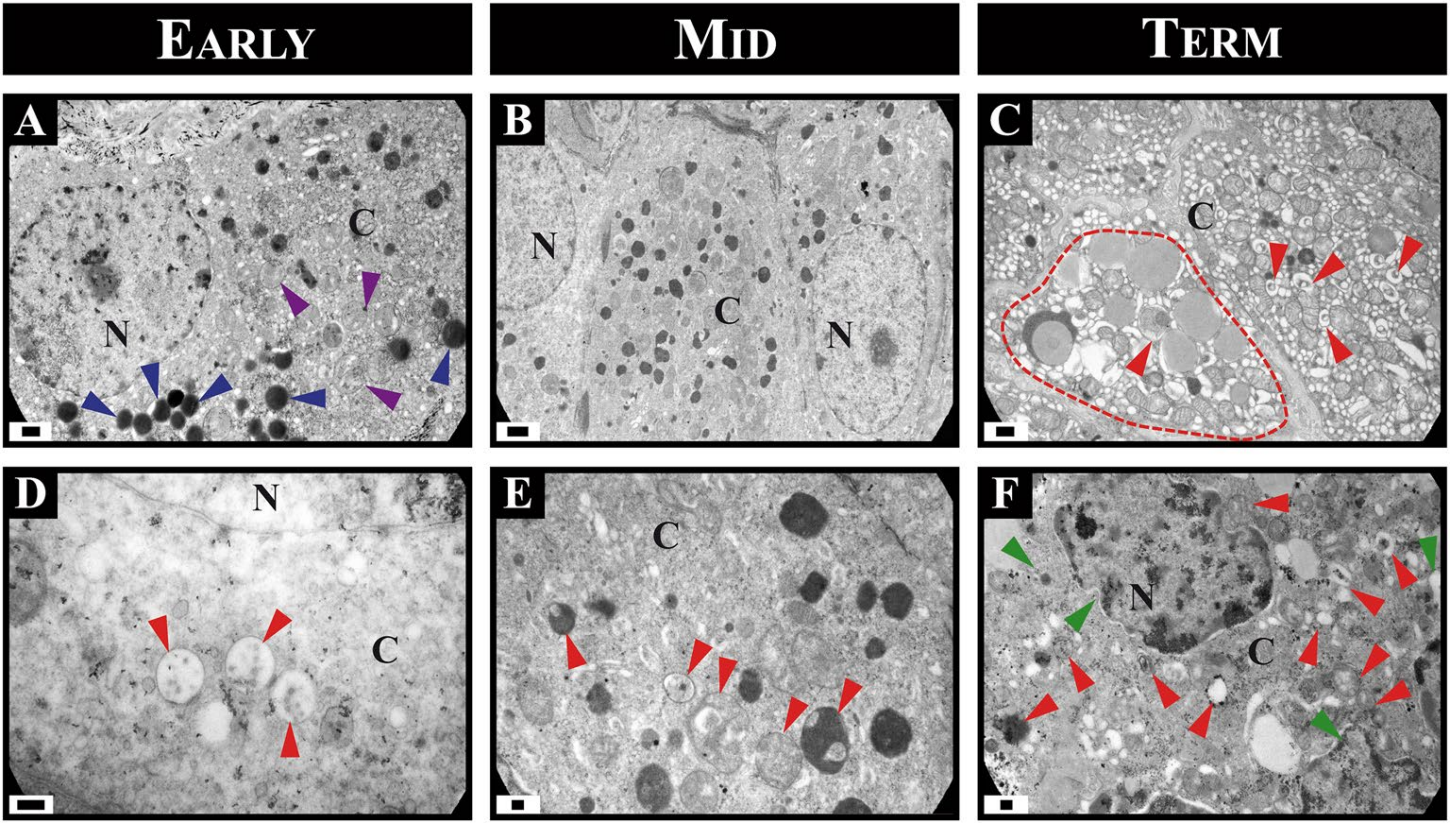
Transmission electron microscopy images of ultrastructural features of autophagy in corpora lutea (CL) of the vizcacha. Healthy CL in early- and mid-pregnancy (**A** and **B**) with homogeneous chromatin in the nucleus, a small number of autophagic vesicles (red arrows), lipid drops (blue arrows), and some mitochondria (violet arrows). Regressed CL (**C**) with altered morphology (green arrows) characterized by an involuted nucleus, condensation of chromatin, and increased number of autophagic vesicles (red arrows) in the cytoplasm of term-pregnant ovaries. Detail of cytoplasm luteal cells of healthy CL with a small number of autophagic vesicles (red arrows) (**D** and **E**) and regressed CL completely filled with autophagic vesicles in its cytoplasm (red arrows) and morphological alterations and blebbing (green arrows) (**F**). C: cytoplasm, N: nucleus. Scale bars: **A**–**C**: 2 μm, **D**–**F**: 0.2 μm.

Transmission electron microscopy (TEM) was used to determine the presence of autophagosomes, autolysosomes, and lysosomes in primary, secondary, and regressed CL during pregnancy. TEM showed differences between degenerative and healthy luteal cells. Healthy luteal cells maintained a close relationship with each other, characterized by a homogeneous cytoplasm containing numerous lipid droplets, well-distributed mitochondria, and sparsely distributed chromatin in the nucleoplasm (Fig. 5A). In primary and secondary CL, a small number of autophagic vesicles were observed within the healthy luteal cells (Fig. 5A, B, C and D).

In contrast, TEM analysis of regressed CL revealed significant morphological alterations in the luteal cells. These changes included the loss and deformation of the rounded cell structure, disrupted cell–cell contacts, and absence of the nucleus, vacuolization, and accumulation of lipofuscin pigments. TEM examination also revealed abundant autophagic vesicles containing highly degraded cytoplasmic material at various stages of maturation in regressed luteal cells (Fig. 5C, F). Regressed luteal cells in term-pregnancy presented double membrane autophagosomes with cytoplasmic material, single membrane autolysosomes with dark and small cytoplasmic remains, and also many lysosomes (Fig. 5C, F). Notably, luteal cells did not exhibit the typical ultrastructural features of apoptosis, such as condensed nuclei, apoptotic bodies, and blebbing of the cytoplasmic membrane (Fig. 5F).

### Apoptosis-related proteins in the ovary of pregnant vizcacha: ACTIVE CASPASE 3 expression

**Figure 6:**
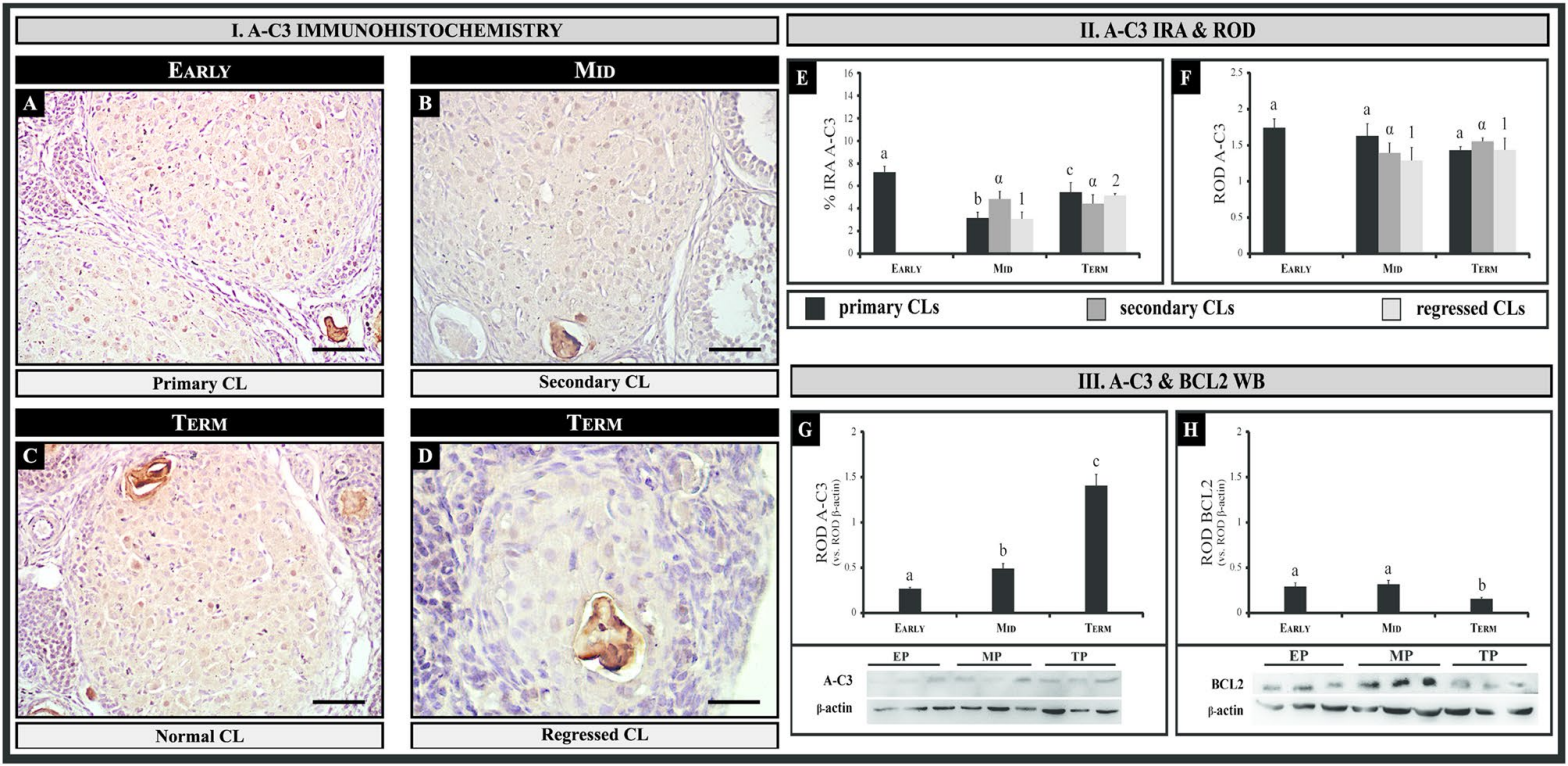
Immunolocalization, relative optical density (ROD), immunoreactive area (IRA), and quantification of protein expression of ACTIVE CASPASE 3 (A-C3) and BCL2 in the ovary of the vizcacha throughout gestation. (**I**) Immunostaining of A-C3 in healthy corpora lutea (CL) in early- (**A**), mid- (**B**), and term-pregnancy (**C**). Immunostaining of A-C3 in regressed CL in term-pregnancy (**D**). II. ROD and IRA of A-C3 (**E**–**F**). (**III)** ROD of protein quantification of A-C3 (**G**) and BCL2 (**H**). β-actin ROD expression was used as a normalizer protein. Representative images of immunoblots for A-C3, BCL2, and β-actin are shown. Values are expressed as mean ± standard deviation. In (**II**), different roman letters indicate significant IRA & ROD differences of primary CLs among groups; different Greek letters indicate significant IRA & ROD differences of secondary CLs among groups; and different numbers indicate significant IRA & ROD differences of regressed CLs among groups (*p* < *0.05*). In III, different Roman letters indicate significant ROD differences among groups (*p* < *0.05*). One-way ANOVA with the Bonferroni post hoc test was used for multiple comparisons. The blots were cut before hybridization with indicated primary antibodies to prevent band overlap. ACTIN was revealed onto a distinct membrane owing to its closely associated molecular weight with BCL2. In some cases, membrane edges are unclear due to the high signal-to-noise ratio of luminescence intensity. Original blots are presented in Supplementary Fig. S6. Scale bar: 50 μm.

In order to correlate autophagy and apoptosis, we analyzed the expression of A-C3 as a late marker of apoptosis in the ovary of pregnant vizcachas. The expression of A-C3 was observed in the nucleus of a few cells from primary, secondary, and regressed CL (Fig. 6). Estimation of the mean percentage of A-C3-immunoreactive CL structures revealed low abundance (< 24%), except in secondary CL with retained oocytes, where the oocyte was positive (> 76%) (Table 2). A-C3 was almost negative in primary CL (except in a few isolated luteal cells). Significant differences were observed in the IRA throughout pregnancy (Fig. 6E). However, ROD did not exhibit significant differences throughout pregnancy (Fig. 6F). Furthermore, A-C3 was observed in degenerative oocytes of secondary CL throughout pregnancy, while luteal cells were almost negative as shown in regressed CL (Fig. 6B–D). No significant differences were observed in the A-C3 IRA and ROD throughout pregnancy for these conditions (Fig. 6E, F). Western blot analysis showed a significant rise in A-C3 expression at mid- and term-pregnancy compared to the early group (Fig. 6G).

### Autophagy as a mechanism of cell survival or cell death in the corpora lutea of the pregnant vizcacha

**Figure 7:**
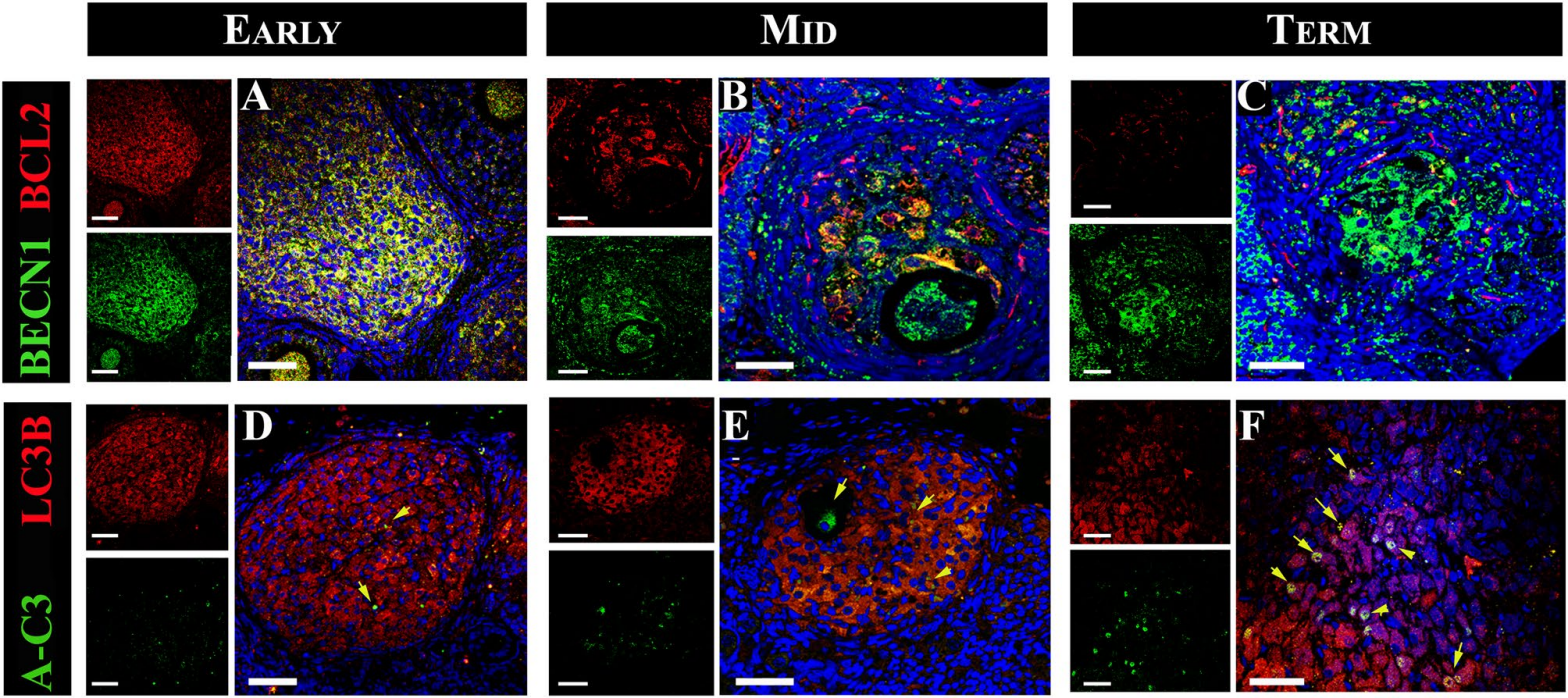
Colocalization of BECN1 with BCL2 and LC3B with ACTIVE CASPASE 3 (A-C3) in healthy and regressed corpora lutea (CL) of the vizcacha throughout gestation. Merged images of BECN1 (cytoplasmic red staining) and BCL2 (cytoplasmic green staining) in primary CL in early-pregnancy (**A**), secondary CL in mid-pregnancy (**B**), and regressed CL in term-pregnancy (**C**). Merged images of LC3B (cytoplasmic red staining) and A-C3 (nuclear green staining) in primary CL in early-pregnancy (**D**), secondary CL in mid-pregnancy (**E**), and regressed CL in term-pregnancy (**F**) (yellow arrows). Yellow cytoplasmic staining indicates co-localization of BECN1 with BCL2 and LC3B with A-C3 in luteal cells of primary (**A** and **D**), secondary (**B** and **E**), and regressed CL (**C** and **F**). All images show DAPI nuclear staining (blue) Scale bar: 50 μm.

To further investigate the apoptotic process in CL with autophagic flux, the colocalization of A-C3 and LC3B was analyzed. The majority of luteal cells did not exhibit simultaneous expression of A-C3 and LC3B (Fig. 7). On the other hand, LC3B expression was detected in the majority of luteal cells. Interestingly, a few luteal cells that exhibited LC3B positivity also showed nuclear signals for A-C3 (Fig. 7).

Furthermore, to evaluate cell survival in CL, the colocalization of the anti-apoptotic protein BCL2 and BECN1 was analyzed. In primary and secondary CL, we observed simultaneous expression of both BECN1 and BCL2 proteins (Fig. 7A, B). Although BCL2 and BECN1 were expressed in secondary CL, in some cases no colocalization of both proteins was detected (Fig. 7B). BECN1 was detected in some luteal cells and oocytes, while BCL2 was detected in luteal and theca cells (Fig. 7B). In regressed CL, only BECN1 expression was found. Western blot analysis demonstrated significant increases in BECN1 and BCL2 expression during early- and mid-gestation. However, at the end of gestation, there was a decrease in BCL2 expression (Fig. 7H).

## Discussion

Autophagy regulates tissue homeostasis, serving as an alternative pathway to apoptosis or as a stress adaptation mechanism to prevent cell death in the life cycle of mammals^52^. In the context of ovarian function, it is a significant process in physiological and pathological conditions^53^, particularly associated with follicular atresia and CL regression^50,53–56^. Notably, the present study reveals that autophagy could serve as a dual mechanism promoting cell survival in healthy primary and secondary CL, while also serving as a cell death mechanism in regressed CL during pregnancy.

In order to maintain optimal embryonic development and sustain the lengthy five-month gestation period of the pregnant vizcacha, both the primary corpus luteum and the subsequent set of secondary corpora lutea employ basal autophagy. This mechanism provides essential nutrients and energy, thereby facilitating the sustenance of luteal cell homeostasis and viability. Our findings suggest that autophagy is potentially pivotal in regulating CL survival during pregnancy, as evidenced by the concurrent expression of BECN1 and BCL2, along with the presence of a limited number of autophagic vacuoles in healthy primary and secondary CL throughout pregnancy. The interplay between autophagy and apoptosis is governed by the interaction of the BH3 binding domains of BECN1 and BCL2^38,57^. In this study, the co-expression of BECN1 and BCL2, alongside the low levels of A-C3 in healthy primary and secondary CL, suggests the involvement of autophagy in maintaining cellular homeostasis and luteolysis in the ovary of pregnant vizcacha. In this regard, it has been suggested that at basal levels of autophagy (pro-survival), the BECN1-BCL2 complex interaction occurs; however, the dissociation of the complex leads to an increase in autophagy, which can trigger cell death depending on the cellular context^58^. In the ovary of the vizcacha, we have previously demonstrated a high expression of BCL2 in CL during pregnancy, consistent with secretory activity for the luteotropic markers^49,59^. Thus, this could prevent BECN1/BCL2 dissociation to maintain luteal cell survival^57,58,60,61^.

The present investigation reveals novel findings regarding the manifestation of autophagy in vizcachas CL at varying degrees of intensity during both development and regression, thus implying its possible role in regulating the lifespan of CL. During early gestation, primary CL exhibited low levels of BECN1, LC3B I-II, SQSTM1, LAMP1, and A-C3, alongside few autophagic vesicles and high levels of BCL2. Notably, post-ovulation at mid-pregnancy displayed a significant increase in autophagic proteins and high levels of P4 and BCL2, correlating with the increase in both the size and number of healthy primary and secondary CL towards the end of gestation. Furthermore, autophagic flux was maintained. Western blot analysis revealed a higher expression of A-C3 at term pregnancy, although it is likely associated with an increase in the number of atretic follicles rather than the CL, as almost no coexpression of A-C3 with LC3B was observed. These results collectively suggest that autophagy may contribute to the luteogenesis in the ovaries of vizcacha during pregnancy. Moreover, autophagy was found to be positively correlated with lipid droplet accumulation in luteal cells, indicating its role in steroidogenesis. Specifically, autophagy-related proteins have been confirmed to play a role in lipid droplet formation and function within non-reproductive tissues^62,63^. Additionally, the middle of the luteal phase represents the highest steroidogenic activity, as indicated by the greatest production of P4^40,46,64^. Therefore, at this developmental stage of the vizcacha CL, autophagy may influence the luteal lipid metabolism as observed by TEM. However, further work is needed to understand the interaction between lipid droplets and autophagosome components. To summarize, this evidence emphasizes the possible role of autophagy in regulating the survival of luteal cells.

Regarding the regression of CL, some authors have proposed apoptosis as the underlying mechanism for luteal cell death^8^. However, the topic of apoptotic processes in the CL is a subject of debate when considering various mammalian species. Hence, it is plausible that apoptosis may not be the sole mechanism involved in CL regression. Currently, there remains a dearth of research investigating the involvement of autophagy in CL regression within mammals^64^. It has been reported that the morphological characteristics of CL regression suggest that autophagy is the cause of luteal cell death in primate ovaries and pregnant rats^65–67^.

At the end of gestation of the vizcacha, specifically during the late luteal phase wherein the three varieties of CL coexist, we have documented a limited number of regressing CL. Within these structures, we have identified the presence of autophagic flux, an abundance of autophagosomes, autolysosomes, and lysosomes. Additionally, we have observed a high expression of autophagic proteins in comparison to healthy CL. On the other hand, the meager expression of A-C3 observed in the majority of luteal cells suggests that autophagy, and not apoptosis, is possibly the main mechanism responsible for the death of these cells. This differs from what is found in the retained oocyte of secondary CL, which always expresses high levels of A-C3 indicating the occurrence of apoptosis. In the ovary of term-pregnant vizcachas, there was a significant increase in autophagic and A-C3 proteins, while the expression of BCL2 decreased. Furthermore, colocalization between BECN1 and BCL2 was infrequent, and there was a rise in autophagic vacuoles and loss of morphology exclusively in regressed CL, indicating the involvement of autophagy in CL regression. It has been proposed that low levels of P4 can induce luteal cell death by autophagy due to the loss of the interaction between BECN1 and BCL2^10,68–70^. In line with this, a decrease in serum levels of P4 at the end of pregnancy is observed in female vizcacha^40^. Our previous report indicated that pro-apoptotic BAX protein was detected in isolated luteal cells of gestating and postpartum females, and apoptosis-associated DNA fragmentation, detected by TUNEL, was scarce and correlated with BAX detection in luteal cells^49^. However, it is possible that proteins associated with apoptosis may not have been strongly detected because it is not the main mechanism involved in CL regression in vizcacha. Instead, autophagy appears to play a more important role in this context.

In this study, we have assessed the lifespan and regression of corpora lutea, taking into account their morpho-histological characteristics (CL size and cell structure), serum estrogen and P4 levels, and the expression of autophagic and apoptotic proteins.

Previous work of our group supports the possible correlation between morphology and functionality raised in this work. Fraunhoffer et al.^40^, studied in depth the ovarian dynamics of *L. maximus* and its correlation with the hormonal profile during gestation, analyzing serum levels of P4, 17b-estradiol, 4D-androstenedione, luteinizing hormone (LH) and follicle stimulating hormone (FSH), as well as the ovarian distribution and expression of their receptors. On the other hand, Cortasa et al.^59^ evaluated the expression of steroidogenic enzymes such as StAR, 3β-HSD, 20α-HSD in CLs throughout gestation. Taken together, these works have demonstrated the constant expression of estrogen, P4 and LH receptors, as well as the presence of steroidogenic enzymes, such as StAR, 3β-HSD and 20α-HSD, in CLs throughout the gestational period.

Our findings indicate sustained activity of CLs during gestation, supported by comparable labeling intensity for luteotropic markers. Although primary CLs exhibit significantly larger sizes than secondary CLs, both show structural disorganization and a decrease in secretory characteristics toward the later stages of gestation. Notably, no substantial DNA fragmentation was detected in luteal cells during the entire gestation period^49,59,71^.

In the future, we propose to deepen our knowledge by evaluating the behavior of autophagy under conditions of pharmacological modulation of the reproductive physiology of the vizcacha.

## Conclusions

The current investigation displays, for the first time, evidence of a binary death/survival autophagy function in the mature ovary of the vizcacha during gestation. The evidence shows various levels of intensity during t CL development and regression, indicating a possible role in the regulation of CL lifespan. In healthy primary and secondary CLs; autophagy is proposed to promote luteal formation and function. Conversely, in the few regressed CLs, autophagy may contribute to the demise of luteal cells. These findings contribute the understanding of autophagy in CL, highlighting its diverse confirmed and potential functions within the female reproductive system.

To facilitate the substantial polyovulation observed in the vizcacha during the subsequent reproductive cycles, the proposed mechanism of autophagy-induced cell death possible serves to eliminate cellular debris and cells that are no longer necessary. This cleaning process begins at the end of gestation and persists in both postpartum and non-pregnant ovaries^50^. Such a process creates the space and material necessary for the growth and maturation of new follicles entering the expanding follicular cohort, thereby ensuring the continuous occurrence of substantial folliculogenesis and polyovulation in the ovary of the vizcacha.

## Materials and methods

### Animals and tissue collection

Over two consecutive breeding seasons, adult pregnant female plains vizcachas were captured using live traps located at the entrance of burrows from a natural resident population at the Estación de Cría de Animales Silvestres (ECAS), Ministry of Agriculture, Villa Elisa, Buenos Aires province, Argentina (34° 51′ 0″ S, 58° 6′ 37″ W). The Ministry of Agriculture Authority of the Buenos Aires Province Government approved the capture of animals under a cooperative agreement established between Universidad Maimónides and the Agriculture Department of Buenos Aires Province. During the main annual breeding season, which spans from April to September, 24 pregnant females were captured at three different time points, encompassing early- (EP, from late March or early April, n = 8), mid- (MP, from early June, n = 8), and term-pregnancy (TP, from late August or early September, n = 8).

In our study, we used a method referred to as “E” for determining sample size. This method, known as the “resource equation” method, relies on the principle of diminishing returns^72^. It is used when no assumptions can be made about the real sample size. We used this method because, in this study, the animals are from the field; they represent wild specimens from a natural resident population in a breeding station whose number of individuals is constantly fluctuating, making it very difficult to determine the total number of resident animals at any given time. On the other hand, our ability to obtain animals is limited, as the number of captures is regulated by the Buenos Aires Government. This regulatory framework allows us to capture a maximum number of animals per year. In addition, as these animals are captured in the field and have seasonal reproductive cycles, there is a narrow range of months suitable for trapping. The number of animals that can be harvested during these months depends on the capture efforts made, which in turn are conditioned by the permits granted, population fluctuations of the species, weather conditions, and other factors that may limit capture efficiency.

The “E” value represents the degrees of freedom of analysis of variance (ANOVA). A sample size ensuring an “E” value between 10 and 20 is deemed adequate. If the value of “E” is below 10, increasing the sample size may enhance the likelihood of obtaining significant results. Conversely, if it exceeds 20, additional samples are unlikely to yield significant outcomes. The calculation of “E” is expressed as the total number of animals (24) minus the total number of groups (3). In our study, “E” is determined as (8 animals per group × 3 groups) – 3 groups = 21, indicating an appropriate sample size for the experiment’s requirements.

The reproductive status of adult pregnant females was assessed on the basis of the weight of animals (> 2.5 kg), time of capture (April to September) according to the natural reproductive cycle described by *Llanos and Crespo*^73^, our own previous field expertise^46,71,74–76^, examination of fetuses, previous description on implantation and embryo development^42,44^. Following capture, the females were transported to the laboratory. After a 3-day resting time, the animals were weighed and anesthetized by the intramuscular administration of 0.4 mg/kg body weight of ketamine chlorhydrate 100 (Holliday Scott S.A., Buenos Aires, Argentina) and 0.6 mg/kg body weight xylazine chlorhydrate 50 (Richmond Laboratories, Veterinary Division, Buenos Aires, Argentina). Blood samples were taken by cardiac puncture with a heparinized syringe, left to rest at room temperature until serum separated, and centrifuged at 3000 rpm for 15 min at room temperature. Recovered serum was stored at − 20 °C until used. After bleeding, animals were humanely sacrificed by means of intracardiac injection of Euthanyl (0.5 ml/kg body weight, sodium pentobarbital, sodium diphenylhydantoin, Brouwer S.A., Buenos Aires, Argentina). The number and location of implanted embryos in each horn were recorded after exposing the uterine horns. Furthermore, the size of the embryo sacs, as well as the cephalocaudal embryo length, was measured using a Vernier caliper to assess the embryonic developmental stage^41^. The embryos situated nearest the cervix, the only ones that complete development to term, were used because all anterior implanted embryos in each uterine horn are subjected to a natural process of resorption^41,44,45^. Table 3 summarizes the gestational characteristics of EP, MP, and TP females. Finally, the ovaries were dissected and examined visually with the naked eye for hemorrhagic follicles (Table 3). One ovary obtained from every female specimen was fixed in cold 4% neutral-buffered para-formaldehyde (PFA) for 24 h before further processing. The other ovary was divided into two halves for the purposes of electron microscopy and western blot analysis, respectively (see below). The study was carried out in compliance with the ARRIVE guidelines. Experimental protocols were reviewed and endorsed by the Institutional Committee on the Use and Care of Experimental Animals (CICUAE, Universidad Maimónides, protocol number 9971/64). The handling and killing of animals were conducted in accordance with the guidelines published in the National Institutes of Health (NIH) guide for the care and use of laboratory animals^77^.

**Table 3:** Gestational characteristics of pregnant female vizcachas used in this study.

Stage of pregnancy	Date of capture	Average female weight (g)	E2 serum levels (*p*g/ml)	P4 serum levels (ng/ml)	Cephalocaudal embryo size (mm)	Ovulatory stigmata
Early	April	2814 ± 626	24.00 ± 8.00	6.52 ± 1.69	9.52 ± 4.28	No
Mid	June	3387 ± 550	45.59 ± 10.31	18.75 ± 8.46	109.36 ± 10.78	Yes
Term	August	2750 ± 300	54.72 ± 19.25	8.95 ± 5.73	140.57 ± 19.26	No

Values are expressed as mean ± standard deviation. E2: 17β estradiol; P4: progesterone.

### Enzyme-Linked Immunosorbent Assay (ELISA) assay.

To determine the serum levels of 17β estradiol (E2) and P4, the ELISA kits EIA263 and EIA1561, respectively (DGR Int.), were used following the manufacturer’s recommendations. A direct immunoassay on a solid phase was performed, with a detection range of 16–2000 pg/ml E2 and 0.18–40 ng/ml P4. The solution absorbance was analyzed at 450 nm using a microplate spectrophotometer (µQuant, Bio-tek Instruments Inc.) and was found to have an inverse correlation with E2 and P4 sample concentration. The E2 and P4 sample concentrations were carried out using calibration curves provided by the manufacturer.

### Ovarian histology and corpora lutea semi-quantification

PFA-fixed ovaries were dehydrated through a graded series of ethanol (50%, 70%, 96%, and 100%), then embedded in paraffin, serially sectioned at 4 μm thickness, and mounted onto cleaned coated slides. Sections were dewaxed in xylene (Sigma Chemical Co., St. Louis, MO, USA) and re-hydrated through a series of decreasing concentrations of ethanol. At least 3 slides of each specimen were stained with hematoxylin–eosin for general histology inspection of the ovulatory status and CL development. Hematoxylin–eosin stained ovary sections were examined to determine the relative abundance of primary, secondary, and regressed CL, as previously described^40,46,49,50^. Semi-quantification of CL was performed in the ovaries of 15 animals, 5 individuals at each pregnancy time-point, using 3 sections per ovary separated by 300 μm each to prevent any potential counting of the same CL. CLs were classified as previously proposed by Fraunhoffer et al.^40^. Secondary CLs were identified as luteinized unruptured follicles containing a retained oocyte, while primary CLs were characterized as larger in size and lacking a retained oocyte. Any CL exhibiting signs of regression, such as loss of morphology, shrinkage, decreased vascularity, and structural disorganization, were classified accordingly. Primary, secondary, and regressed CLs were counted to estimate their relative abundance and expressed as mean CL per mm^2^ of ovarian tissue. Counting was performed at 10X magnification and representative images were captured with an optic microscope (BX40, Olympus Optical Corporation, Tokyo, Japan) fitted with a digital camera (390CU 3.2 Megapixel CCD Camera, Micrometrics, Spain) and the image software Micrometrics SE P4 (Standard Edition Premium 4, Micrometrics, Spain). CL major axes were determined using Image-Pro Plus software (Image Pro Plus 6, Media Cybernetics Inc, Bethesda Maryland, USA). The remaining consecutive serial-sectioned slides to those used for CL counting were stored at room temperature until used for immunohistochemistry or immunofluorescence (8 animals per experimental group).

### Immunohistochemistry

De-waxed and re-hydrated ovarian sections were placed in sodium citrate buffer (10 mM sodium citrate, 0.05% Tween-20, pH 6.0) for heat-induced epitope retrieval for 1 min in a water bath at 100 °C, then sections were treated with 3% H_2_O_2_ for 20 min at room temperature to block the activity of endogenous peroxidase, followed by incubation in a blocking solution containing 10% bovine fetal serum in phosphate saline buffer (PBS) (pH 7.4) for 30 min at room temperature. Blocked sections were incubated overnight at 4 °C with specific primary antibodies: rabbit anti-LAMP1 IgG (1:500, ab24170, Abcam, Cambridge, UK), rabbit anti-LC3B IgG (1:500, ab48394, Abcam, Cambridge, UK), rabbit anti-Beclin 1 (BECN1) IgG (1:500, ab62472, Abcam, Cambridge, UK), rabbit anti-Sequestosome 1 (SQSTM1) IgG (1/250, PA5-27247, Invitrogen, Massachusetts, USA**)** and rabbit anti-Active capase 3 (A-C3) IgG (1:300, ab2302 and ab13847, Abcam, Cambridge, UK) followed by a 1-h incubation in biotinylated anti-rabbit IgG (1:200 dilution) (Vector Labs, Peterborough, UK), washed in PBS, incubated with an avidin–biotin complex (ABC Vectastain Elite Kit, Vector Laboratories, Burlingame, CA, USA), further washed in PBS and incubated for 30 min with 1:100 diluted streptavidin-peroxidase complex (ABC kit, Vector Labs, Peterborough, UK). The reaction was visualized with DAB (SK-4100, DAB Kit, Vector Laboratories, Burlingame, CA, USA). Finally, sections were dehydrated through a graded series of ethanol (70, 96, and 100%), cleared in Neo-Clear (Merck, Darmstadt, Germany), and coverslipped. Microscope images of the immunoreactivity were captured with a light microscope (BX40, Olympus Optical Corporation, Tokyo, Japan), fitted with a digital camera (390CU 3.2 Megapixel CCD Camera, Micrometrics, Spain) and the image software Micrometrics SE P4 (Standard Edition Premium 4, Micrometrics, Spain). All images were taken the same day under the same light to avoid external variations. Staining for each antibody was repeated at least four times in separate assays for each specimen, using a minimum of three slides per assay. Proximal, medial, and distal sections of the whole organ were used. All antibodies were screened in serial sections on the same slide.

For the assessment of immunoreactivity levels for each marker, a semi-quantitative analysis was conducted using the Image Pro Plus software (Image-Pro Plus 6, Media Cybernetics Inc.). The relative optical density (ROD) and immunoreactive area (IRA) were determined. A total of 10 images of primary, secondary, and regressed CL were evaluated for each gestation group at a magnification of 10X to ensure comprehensive visualization of the entire structure in each image. The CL was delineated, and ROD was quantified. Background regions without immunoreactivity were considered for normalization of the positive signal density. The IRA was calculated for each CL, representing the percentage of the positive labeled area over the total CL area. All images were captured on the same day under standardized lighting conditions to minimize external variations. Adobe Photoshop CS5 software (Adobe Systems Inc.) was employed for the adjustment of brightness and contrast. Statistical analysis was performed using the GraphPad Prism program (version 4.0 for Windows, GraphPad Software Inc.). Results were expressed as mean ± standard deviation (SD). For comparisons among multiple data groups, analysis of variance (ANOVA) was employed, followed by Bonferroni post hoc test for multiple comparisons. Statistical significance was defined as *p* < 0.05.

### Immunofluorescence with confocal microscopy

For the analysis of protein colocalization, dewaxed and re-hydrated sections was subjected to a first blocking of endogenous IgG with a 15 μl solution of normal goat or rabbit blocking serum, depending on the primary antibody (Vectastain Elite ABC Kit (Vector Laboratories), for 45 min at room temperature. Immunoreactivity was detected by incubating the slides overnight at 4 °C with specific primary antibodies: rabbit anti-LAMP1 IgG (1:500, ab24170, Abcam, Cambridge, UK), rabbit anti-LC3B IgG (1:500, ab48394, Abcam, Cambridge, UK), rabbit anti-BECN1 IgG (1:500, ab62472, Abcam, Cambridge, UK), rabbit anti-SQSTM1 IgG (1/250, PA5-27,247, Invitrogen, Massachusetts, USA**),** rabbit anti-A-C3 IgG (1:300, ab2302 and ab13847, Abcam, Cambridge, UK), rabbit anti-BCL2 (1:500, ab7973, Abcam, Cambridge, UK). After further washing in PBS-Tween 20, slides were incubated with secondary antibodies Alexa-Fluor 488 coupled goat anti-rabbit IgG (Invitrogen Corp.), and Alexa-Fluor 555 coupled donkey anti-rabbit IgG (Invitrogen Corp.) or Alexa- Fluor 488 coupled donkey anti-rabbit IgG (Invitrogen Corp.) for 1 h at room temperature. Sections were rinsed in PBS-Tween 20 and a second block of endogenous IgG was performed. Sections were incubated with the second primary antibody and secondary antibodies conjugated with a fluorochrome in a humid and dark chamber overnight at 4 °C. Counterstaining with DAPI (100 ng/ml in PBS) was performed for 10 min at room temperature. Colocalization of autophagic and apoptotic proteins was visualized by immunofluorescence using a Nikon C1 Plus Laser microscope (Nikon Inverted Research Microscope Eclipse Ti, Nikon Corp., Tokyo, Japan) and images were analyzed with the EZ-C1 software (EZ-C1 Software v3.9, Nikon Ltd ., London, UK).

### Semi-quantification of autophagic and apoptotic markers in ovarian structures

The semi-quantification of positive CL for BECN1, LAMP1, LC3B, SQSTM1, and A-C3 markers was conducted by enumerating both positive and negative structures for each marker in a total of 3 sections per animal for each gestational time point, utilizing randomly-selected slides. Immunohistochemistry-treated sections were examined under 20× magnification using an Olympus B×40 microscope (Tokyo, Japan) to identify reactive structures. Results were expressed as mean ± SD. For comparisons among multiple data groups, ANOVA was employed, followed by Bonferroni post hoc test for multiple comparisons. Statistical significance was defined as *p* < 0.05.

### Transmission electron microscopy

In order to analyze autophagic vacuoles, half of an ovary from each female was sectioned in blocks of 1 × 1 mm thick with a scalpel blade under a magnifying glass Olympus SZX7, fixed in cold 4% paraformaldehyde, 0.25% glutaraldehyde, in 0.1 M PBS (pH 7.4, 72 h), and transferred to fresh PBS. Sections were subsequently post-fixed in 2% osmium tetroxide containing 1.5% potassium ferricyanide for 1 h, dehydrated in graded alcohols and propylene oxide, and embedded in FlukaDurcupan (Sigma). Semi-thin Sects. (0.50 μm thick) were obtained using an ultramicrotome (Reichert Jung Ultra cut E) and counterstained with toluidine blue for observation in a light microscope. The area of interest was selected and ultrafine cuts (70–90 nm thick) were made with the Ultra microtome (Reichert Jung Ultracut E). The cuts were mounted on copper grids and contrasted with uranyl acetate and lead citrate (Reynolds method). The cuts were observed in the Zeiss 109 transmission electron microscope attached to the Gatan 1000 digital camera.

### Western blot

Small ovarian fragments were immersed in lysis solution (3 ml/g tissue) containing RIPA buffer (1% igepal, 0.5% sodium deoxycholate, 0.1% sodium dodecyl sulfate) with protease inhibitors (200 mM phenylmethylsulfonyl fluoride, 100 mM sodium orthovanadate, 10 μM leupeptin, 1 μM pepstatin, and 10 μM aprotinin), homogenized in an ice-bath during 30 s at high speed with the Bio-Gen PRO200 homogenizer (PRO 200, Pro Scientific Inc, Oxford, USA) and centrifuged at 10,000 rpm for 10 min at 4 °C. Protein content was determined with Bradford assay (Bio-Rad Protein Assay, Bio-Rad Laboratories Inc.EE.UU, California). Total proteins (15 μg) were separated by one-dimensional 12% SDS-PAGE and then transferred onto PVDF membranes (Immobilon-P Transfer membrane, Millipore, Bedford, USA). Membranes were blocked for 1 h in PBS with 5% non-fat dry milk and incubated with specific primary antibodies: rabbit anti-LAMP1 IgG (1:500, ab24170, Abcam, Cambridge, UK), rabbit anti-LC3B IgG (1:500, ab48394, Abcam, Cambridge, UK), rabbit anti-BECN1 IgG (1:500, ab62472, Abcam, Cambridge, UK), rabbit anti-SQSTM1 IgG (1/250, PA5-27,247, Invitrogen, Massachusetts, USA**),** rabbit anti-A-C3 IgG (1:300, ab2302 and ab13847, Abcam, Cambridge, UK), and rabbit anti-BCL2 (1:500, ab7973, Abcam, Cambridge, UK). Then, samples were incubated for 2 h with goat anti-rabbit IgG (H + L) horseradish peroxidase-conjugated secondary antibody (1:2000, Sigma, Saint Louis, MO, USA). The immunoreactive product was visualized using the enhanced chemiluminescence system (ECL plus or prime GE, Amersham, Fairfield, CN, USA). Membranes were scanned with ImageQuant 350 (GE Healthcare BioSciences AB, Uppsala, Sweden) and dot-blots analyzed with Image-Pro Plus software (Image-Pro 230 Plus 6, Media Cybernetics Inc., Bethesda, Maryland, USA). The estimation of band size was performed using a pre-stained protein ladder (PageRuler, Fermentas UAB, Vilnius, Lithuania) as molecular weight markers. Protein expression was normalized to β-actin (Sigma, Saint Louis, Missouri, USA).

### Statistical analysis

Data are presented as mean ± SD and statistical analysis was performed with Prism 6.0 (GraphPad Software Inc., San Diego, California, USA). One-way ANOVA with the Bonferroni post hoc test was used for multiple comparisons. Differences were considered statistically significant when *p* < 0.05.

### Supplementary Information

Supplementary Information.

## Data Availability

The datasets used and/or analyzed during the current study available from the corresponding author on reasonable request.
