# Supplementary material for: Autophagy favors survival of corpora lutea during the long-lasting pregnancy of the South American plains vizcacha, Lagostomus maximus (Rodentia, Caviomorpha)
Source: Sci Rep. 2024 May 16;14:11220. doi: 10.1038/s41598-024-61478-5 (PMC11099099; doi:10.1038/s41598-024-61478-5)
Supplement: Supplementary file 1 — Supplementary Information. [file 41598_2024_61478_MOESM1_ESM.pdf]

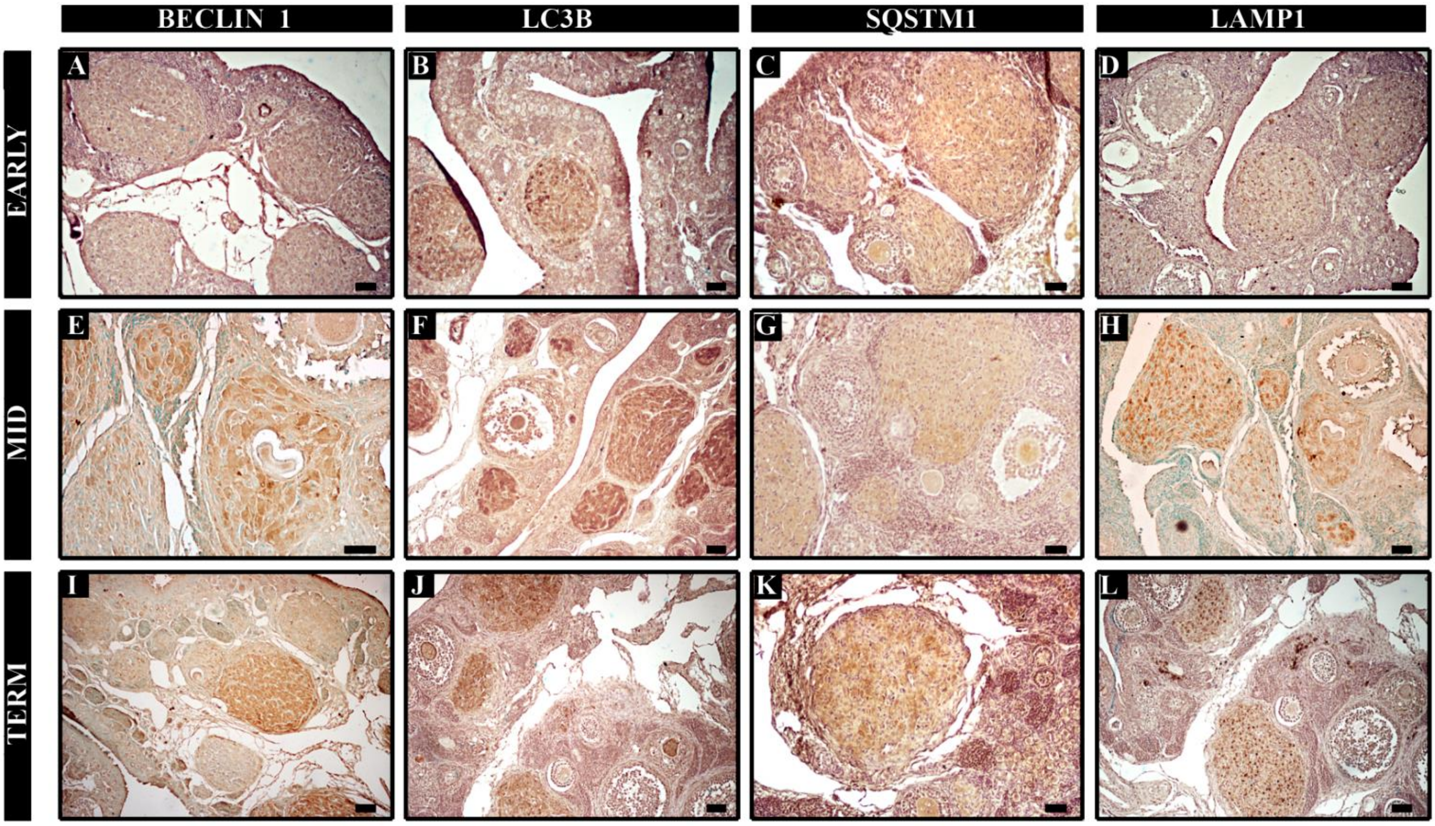

Supplementary Figure 1. Panoramic Immunolocalization of autophagy-related proteins in corpora lutea (CL) of the vizcacha throughout pregnancy. Immunostaining of BECN1 (A, E, I), LC3B (B, F, J), SQSTM1 (C, G, K), and LAMP1 (D, H, L). Scale bars: 50µm.

A

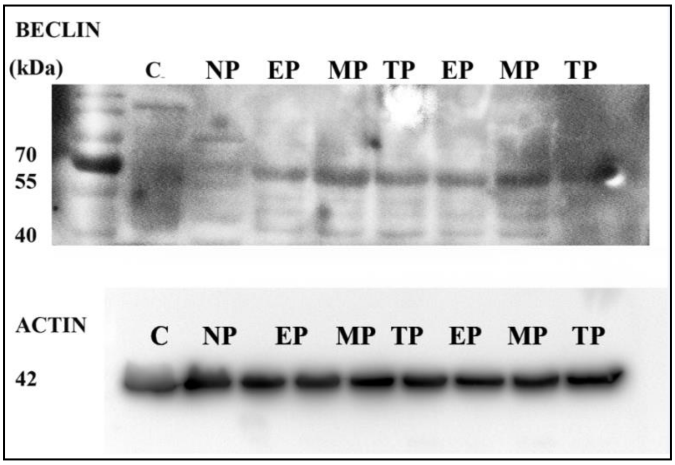

B

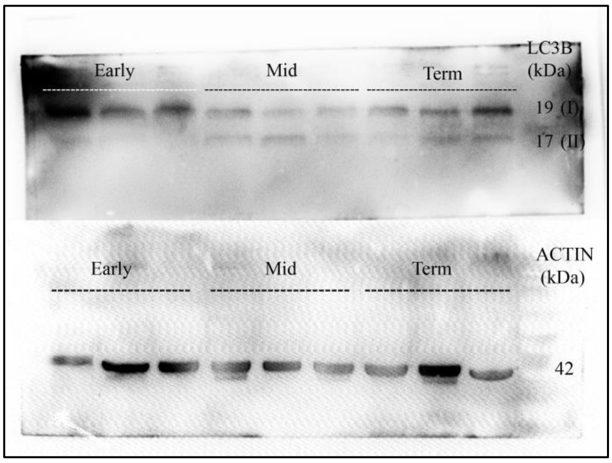

C

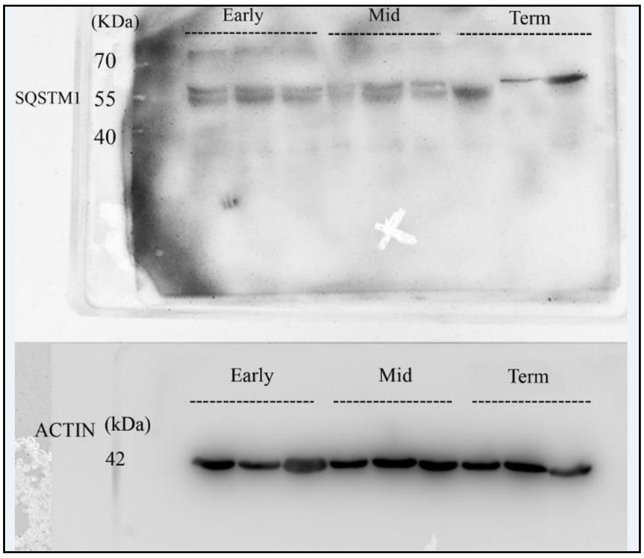

D

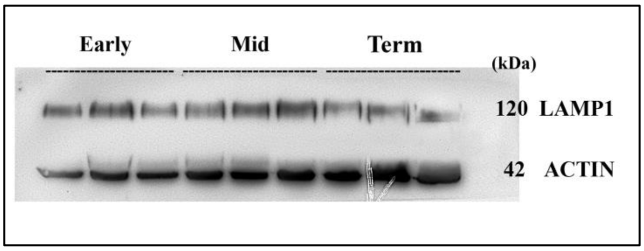

Supplementary Figure 3: Original immunoblots images for(A) BECLIN1, (B) LC3B I-II, (C) SQSTM1, and (D) LAMP1.The blots were cut before hybridization with indicated primary antibodies to prevent band overlap. In some cases, membrane edges are unclear due to the high signal-to-noise ratio of luminescence intensity. C: Control; NP: No pregnant; EP: Early pregnancy; MP: Mid pregnancy; TP: Term pregnancy.

G

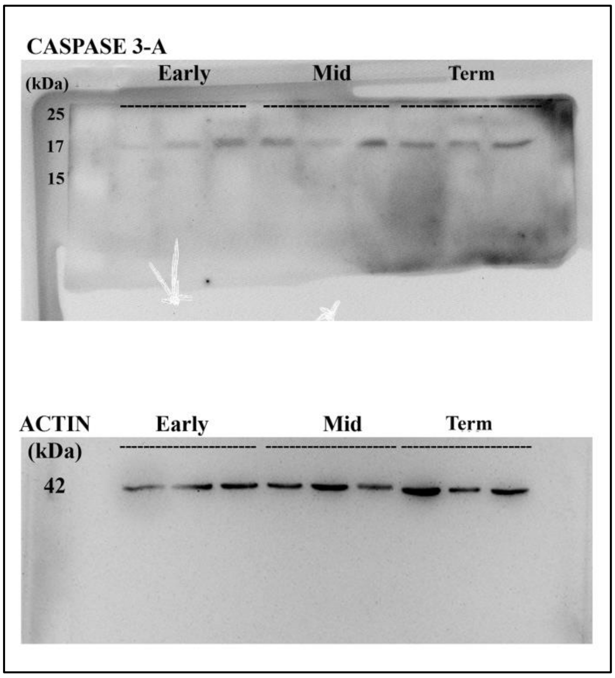

H

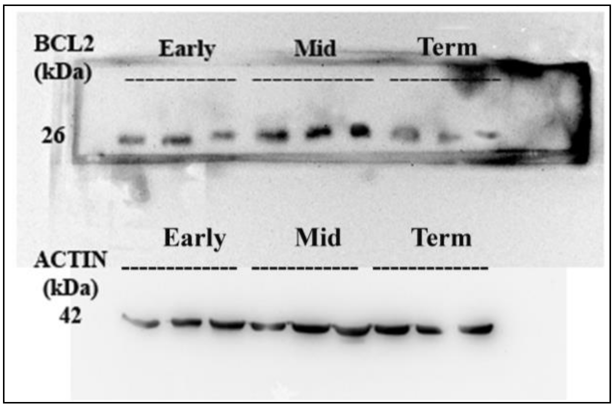

Supplementary Figure 6: Original immunoblots images for (G) CASPASE 3-ACTIVE, and (H) BCL2. All blot membranes were cut into stripes before hybridization with antibodies. In some cases, membrane edges are unclear due to the high signal-to-noise ratio of luminescence intensity.
